# Supplementary material for: Instruments to identify risk factors associated with adverse childhood experiences for vulnerable children in primary care in low- and middle-income countries: A systematic review and narrative synthesis
Source: PLOS Glob Public Health. 2022 Oct 5;2(10):e0000967. doi: 10.1371/journal.pgph.0000967 (PMC10021915; doi:10.1371/journal.pgph.0000967)
Supplement: S1 Table — (DOCX) [file pgph.0000967.s002.docx]

**S1 Table Characteristics of instruments to assess adversities related to poverty in Low- and Middle-Income Countries (LMICs)**

| Author/year | Study aims | Instrument tool/items | Scales/dimension | Indicators | Psychometric properties | Target population/sample size | Country and settings | Rater | Type of study | Mean ±SD for utility measure |
| --- | --- | --- | --- | --- | --- | --- | --- | --- | --- | --- |
| Betancourt et al., 2014 | This study aimed to refine a dimensional scale for measuring psychosocial adjustment in African youth using item response theory (IRT) | African Youth Psychosocial Assessment Instrument (AYPA) - 60 items | Likert scales (1-5)  Psychological | Internalising/externalising behaviours | APAI1 - I listen to others  APAI2 - I play together with others  APAI3 - I have a lot of thoughts  APAI4 - I have constant worries  APAI5 - I have pain all over my body  APAI6 - My brain is not functioning well  APAI7 - I think I am of no use  APAI8 - I think about suicide  APAI9 - I constantly talk about my problems  APAI10 - I sit alone  APAI11 - I share with others  APAI12 - I lose interest in school  APAI13 - I get headaches  APAI14 - I lose my appetite  APAI15 - I feel a lot of pain in my heart  APAI16 - I sit with my cheek in my palm  APAI17 - I cry when I am alone  APAI18 - I do not sleep at night  APAI19 - I am disobedient  APAI20 - I feel cold  APAI21 - I share food and eat with others  APAI22 - I lie down all the time (during the day)  APAI23 - I have lots of worries  APAI24 - I want to be alone  APAI25 - I am easily annoyed  APAI26 - I hold my head  APAI27 - I lose concentration in class  APAI28 - I drink alcohol  APAI29 - I insult my friends  APAI30 - I don't greet people  APAI31 - I help others  APAI32 - I don't think straight  APAI33 - I mutter to myself  APAI34 - I don't trust  APAI35 - I feel I can do nothing to help myself  APAI36 - I fight  APAI37 - I use bad language  APAI38 - I am disrespectful  APAI39 - I misbehave  APAI40 - I am disinterested  APAI41 - I welcome others  APAI42 - I deceive  APAI43 - I am a rough person  APAI44 - I use drugs (like jayi, marijuana)  APAI45 - I cling to elders  APAI46 - I think I do not have a future  APAI47 - I am constantly running around  APAI 48-I don't like noise  APAI49 - I think people are chasing me  APAI50 - I have a fast heart rate  APAI51 - I cooperate with others  APAI52 - I fear being alone  APAI53 - I feel sad  APAI54 - I think of bad things  APAI55 - I am weak  APAI56 - I don't feel like talking  APAI57 - I am forgetful  APAI58 - I cry continuously  APAI59 - I do not care whether I live or die  APAI60 - I respect others | 14–17-year-old children exposed to war (n=667) | Uganda | Social worker | RCTs | 15.3 |
| Jordans, Ventevogel, Komproe, Tol & de Jong, 2008 | This study aimed to validate the instrument in affected zones  cross-cultural | Child Psychosocial Distress Screener (CPDS) - 7 items | Likert scales  (1-4)  Psychological | Maltreatment | 1.1 Did you experience any shocking or disturbing event(s)?  [Burundi probes: witnessing the killing of family members, witnessing the killing between ethnic groups, attacks by rebels]  [Sudan probes: rape; aerial bombings; witnessing the killing of parents; attacks and abductions by Murahalin; having to kill during duty; being abused]  [Sri Lanka probes: bereavement; aerial bombings; accidents; sexual abuse; abduction by 'white van'; clay-bomb explosions; fear of robbers]  [Indonesia probes: bombings, gun shootings, burning houses, witnessing killings and atrocities, attacks by rebels, abductions, displacement]  0 (never) 1 (sometimes) 2 (often)  1.2 Have you been distressed by these events?  0 (not at all) 1 (a little) 2 (a lot)  1.3 Are you distressed or experiencing problems lately (past few weeks)? [Burundi probes: anger or aggression; absent-minded; withdrawn or isolated; loss of interest; sadness; anxiousness; hyperactive]  [Sudan probes: social withdrawal; aggression; hyperactivity; dominating behaviours]  [Sri Lanka probes: sadness; withdrawal; reduced self-care; sleep disturbances; somatic complaints; violent; hyperactive behaviours]  [Indonesia probes: withdrawal; aggression; loss of concentration; bedwetting; sadness; sleep problems] | 7-17 year old children who have experienced armed conflict | Burundian,  Sri Lankan,  Indonesian, and Sudanese  schools | Teacher | RCTs | 10.36(SD=1.52) for Sri Lanka  10.37 (SD=1.42) for Burundi  9.79 (SD=1.40) for Indonesia 12.0 (SD=1.45) for Sudan |
| Van de Hauvel, 2017 | To assess the prevalence and severity of developmental and behavioural disorders on a cohort of children admitted to an in-patient nutritional rehabilitation centre in Malawi | Malawi Developmental Assessment Tool (MDAT) - 136 items | Anthropometric (weight, height, MUAC), physical | Stunting SAM - Severe Acute Malnutrition  **Poverty**  Kwashiorkor and Marasmus  HIV (human immune deficiency virus) |  | 5 yrs (1,513) | Malawi | Hospital | RCTs | Mean SD - 17.2 months |
| Corcoran & Wakia, 2016 | To monitor the wellbeing of children who have lived on the streets | Child Status Index - 12 items | 4-dimension child status scores  Psychological, physical,  psychological | Food security  Nutrition and growth  Abuse and exploitation  Shelter and care  Access to healthcare  Wellness  Education performance and access  Protection | 1. Food and nutrition 2. The child has sufficient food to eat at all times of the year 3. The child is growing well compared to others of his/her age in the community 4. The child has a stable shelter that is adequate, dry, and safe 5. The child has at least one adult (aged 18 or over) who provides consistent care, attention, and support 6. The child is safe from any abuse, neglect, or exploitation 7. The child has access to legal protection services as needed 8. The child is physically healthy 9. The child can access healthcare services, including medical treatment when ill, and preventive care 10. The child is happy and content with a generally positive mood and hopeful outlook 11. The child is cooperative and enjoys participating in activities with adults and other children 12. The child is progressing well in acquiring knowledge and life skills at home, school, job training, or an age-appropriate productive activity 13. The child is enrolled and attends school or skills training or is engaged in age-appropriate play, learning activity, or job | 13-19 year old orphans and vulnerable children due to HIV/AIDS | Ethiopia | Street | Cross-sectional |  |
| World Health Organisation | To devise a measure of quality of life that is both reliable and valid | WHOQOL-BREF | Five-point Likert Scale | Physical  Psychological  level of independence  Environmental | **Domain I Physical**  Pain and discomfort  Energy and fatigue  Sexual activity  Sleep and rest  Sensory functions  **Domain II Psychological**  Positive feelings  Thinking, learning, memory, and concentration  Self-esteem  Bodily image and appearance  Negative feelings  **Domain III Level of independence**  Mobility  Activities of daily living  Dependence on medicinal substances and medical aids  Dependence on non-medicinal substances (alcohol, tobacco, drugs)  Communication capacity  Work capacity  Social relationships  Personal relationships  Practical social support  Activities as provider/supporter  **Domain iv Environment**  Freedom  Physical safety and security  Home environment  Work satisfaction  Financial resources  Health and social care  Accessibility and quality  Opportunities for acquiring new information and skills  Participation in, and opportunities for, recreation/leisure activities  Physical environment: pollution/noise/ tragic climate  Transport domain | Children and adults (n=300) | 17 countries |  | RCTs |  |
| Collings, Valjee & Penning, 2013 | To develop a preliminary validation of the Developmental Trauma Inventory (DTI) | Developmental Trauma Inventory  (DTI) – 36 items | Likert scale (1-5) Psychological | Poverty and violence,  emotional abuse, community assault, domestic assault, poverty, witnessing community violence, witnessing domestic violence, indecent assault, domestic neglect, rape, and domestic injury | My caretakers treated me in ways that made me feel ashamed  I felt unloved at home  Hurtful things were said to me by my caretakers  In my caretaker's eyes, nothing I said was good enough  People in my family called me insulting names  Being punched or kicked by a non-family member  Being physically attacked by a non-family member  A non-family member cutting you with a knife or sharp object  Being hit with a stick or some other object by a non-family member  A non-family member trying to strangle you  A non-family member burning you with a cigarette or flame  Being punched or kicked by a family member  Being physically attacked by a family member  A family member hitting you with a stick or some other object  A family member trying to strangle you  A family so poor there was not enough food to eat  Parents could not afford to send me to the doctor when I was sick  Parents did not earn enough money to support a family  I witnessed physical violence in my home  My parents hurt each other physically when they argued/fought  A family member got medical treatment because of family violence  I saw someone in the community being beaten, stabbed, or shot  I saw someone in the community being killed  I saw someone in the community being assaulted  Someone touched my sex organs when I did not want them to  Someone made you touch their sex organs when you did not want to  Someone kissing or touching you in a sexual way  Someone making you touch them in a sexual way  Spent time away from home and no one cared  No-one made sure I got up in the morning to go to school  I felt no-one cared if I lived or died  Caretakers did not care when I was unwell or in trouble  Unwanted anal sex  Unwanted genital sex  A family member deliberately burnt you with a cigarette/flame  A family member deliberately cut you with a knife | 12-18 year old (n=720)  war and climate associate adversity | South African | Self-administered | RCTs | 15.43 (1.78) |
| ILO - International  Labour Organisation International Program on the Elimination of Child Labour (IPEC) ‐ Geneva, ILO, 2014. | The "Instrument for the Psychosocial Assessment of Child Workers" (IPAC) is a questionnaire that examines 12 domains deemed relevant to working with children | IPAC: Instrument for Psychosocial Assessment for Child Workers - 48 items | Likert scales  (1-4)  Adversity  Child labor  Physical  Social  Psychological | Maltreatment | 1. Are you proud of your work?  2. Do you feel that you have the skills to do your job well?  3. Do you think others appreciate the job you do?  4. Do some people look down on you because of the kind of work you do?  5. Do you feel that your family relies on you and needs your help?  6. Do you feel under pressure to work faster and harder?  7. Do you feel bored because there is not enough to do?  8. Does your family, employers, or others ask too much of you?  9. Do you get bored at work doing the same time for many hours in a row?  10. Do you feel tired because of the long working hours or heavy workload?  11. Do you feel like your work prevents you from doing things you would like to do?  12. Do you feel like if you wanted to, you could choose what to do and what not to do?  13. Does the environment you are working in bother you at all?  14. Are you comfortable with the people you work with?  15. At work, do you feel that people watch you so that you don't get hurt?  16. Do people at work teach you what to do and how to do it?  17. Do you have free time each day to just do what you want?  18. Do you have a lot of energy?  19. Do you generally feel confident?  20. Do you have any difficulties sleeping?  21. Do you have trouble concentrating?  22. Do you feel restless and cannot stay still for a long time?  23. Do you feel sad and like crying?  24. Do you get into fights and quarrel easily?  25. Do you feel lonely?  26. Do you get very angry and often lose your temper?  27. Do you have little appetite or interest in food?  28. Do you find that you forget things?  29. Do you get tension in your body?  30. Do you feel dizzy?  31. Do you feel afraid or nervous?  32. Do you worry and think a lot?  33. Do you think a lot about bad things that have happened to you in the past?  34. Do you think your life will get better someday?  35. Do you think your life is worse than that of other children?  36. Do you think it isn't worth living?  37. Do you have loving support from your family?  38. Is there conflict in your family?  39. Do you feel accepted by other families around here?  40. Do you have one or more good friends that support you?  41. Do people reject or tease you or call you names?  42. Do you play sports or games with friends?  43. Do you feel very different from other children your age?  44. Do you get scolded or criticized or made to feel small or stupid at work?  45. Do you ever get beaten at work?  46. Has anyone at work tried to touch you in a bad way?  47. Have you been severely punished for a mistake you have made at your work?  48. In your day-to-day life, do you feel safe? | 10-18-year-olds (childhood labor)  (n=207) | Cross-cultural | Social worker | RCTs | (SD=12.69)  Mean 30.7 |
| Boyes, Mason & Cluver, 2013 | Validating of HIV Adolescence Stigma Scale | HIV Stigma-by-Association Scale for Adolescents | Questionnaires  7 item scale | Poverty  Distress  HIV stigma | 1. Teased  2. Treated badly  3. Gossiped about  4. Feel different or alone  5. Worry about rejection  6. Avoid making new friends  7. Afraid of me  8. Think I am a bad person  9. Avoid touching me | 11- and 25-year-old children whose families have been affected by HIV/AIDS  (n=723) | Social workers | South Africa | RCTs | M16.90,  SD 2.50 |
| Cluver & Gardner, 2006 | This study aimed to investigate mental health outcomes for urban children living in deprived settlements in Cape Town | Strengths and Difficulties Questionnaire (SDQ) | Adversity | Emotional and behavioural problems, peer and attention difficulties, and pro-social behaviour experiences of violence, hunger, and school attendance | Extreme difficulty concentrating  Very frequent nightmares  Definite lack of close friendship  Very frequent somatic symptoms | 6 to 19-year-old (n=60) children living in poverty | Teacher | South Africa | RCTs | (SD 3.2) |

This table presents all identified instruments in this review to identify Adverse Childhood Experiences associated with poverty in LMICs.
