# Supplementary material for: Instruments to identify risk factors associated with adverse childhood experiences for vulnerable children in primary care in low- and middle-income countries: A systematic review and narrative synthesis
Source: PLOS Glob Public Health. 2022 Oct 5;2(10):e0000967. doi: 10.1371/journal.pgph.0000967 (PMC10021915; doi:10.1371/journal.pgph.0000967)
Supplement: S2 Table — (DOCX) [file pgph.0000967.s003.docx]

S2 Table Risk assessment tool ranking for use in practice using a 10-point checklist for external validation measures

Risk assessment tool Externally validated ? Tested ? Methodology ? Conceptual framework? Feasible for use?

| African Youth Psychosocial Assessment Instrument (AYPA) | + | + | + | + | - | 4/5 |
| --- | --- | --- | --- | --- | --- | --- |
| Child Psychosocial Distress Screener (CPDS) | + | + | + | + | - | 4/5 |
| Malawi Developmental Assessment Tool (MDAT) | + | - | + | + | - | 4/5 |
| Child Status Index | + | + | + | + | - | 4/5 |
| Developmental Trauma Inventory  (DTI) | + | + | + | + | - | 4/5 |
| IPAC: An instrument for Psychosocial Assessment for Child Workers | + | + | + | + | - | 4/5 |
| HIV Stigma-by-Association Scale for Adolescents | + | + | + | + | + | 5/5 |
|  |  |  |  |  |  |  |
| Strengths and Difficulties Questionnaire (SDQ) | + | + | + | + | - | 4/5 |
| WHO-BREFF | + | + | + | + | + | 5/5 |
