# Supplementary material for: Instruments to identify risk factors associated with adverse childhood experiences for vulnerable children in primary care in low- and middle-income countries: A systematic review and narrative synthesis
Source: PLOS Glob Public Health. 2022 Oct 5;2(10):e0000967. doi: 10.1371/journal.pgph.0000967 (PMC10021915; doi:10.1371/journal.pgph.0000967)
Supplement: S3 Table — (DOCX) [file pgph.0000967.s004.docx]

S3 Table Risk assessment tool ranking for use in practice using a 10-point checklist for internal validation measures

| Risk assessment tool | Does the tool follow a protocol? | How were candidate variables selected? | How are variables weighted? | How were other parameters selected? | Has internal validation been done? | Ranking |
| --- | --- | --- | --- | --- | --- | --- |
| African Youth Psychosocial Assessment Instrument (AYPA) | NR | Purposive sampling | Item Response Theory (IRT) | Structural Equation Modelling (SEM) | + | 4/5 |
| Child Psychosocial Distress Screener (CPDS) | + | NR | Confirmatory Factor Analysis (CFA) | Logistic regression | + | 4/5 |
| Malawi Developmental Assessment Tool (MDAT) | + | Quota sampling technique | Confirmatory Factor Analysis (CFA) | Logistic regression and reliability using kappa statistics | + | 5/5 |
| Child status Index | NR | Random sampling | Spearman Rank | correlation coefficients | + | 4/5 |
| Developmental Trauma Inventory (DTI) | NR | Purposive sampling | Confirmatory Factor Analysis (CFA) | Logistic regression and reliability using kappa statistics | + | 5/5 |
| IPAC: An instrument for Psychosocial Assessment for Child Workers | + | Purposive sampling | Theoretical model | Logistic regression | + | 5/5 |
| HIV Stigma-by-Association Scale for Adolescents | + | Purposive sampling | Exploratory factor analyses | Correlation coefficients  Kappa  Cronbach's alpha) | + | 5/5 |
| Strengths and Difficulties Questionnaire (SDQ) | NR | Purposive sampling | Theoretical model | t-tests and Pearson's chi-square | + | 5/5 |
| WHO-BREFF |  |  |  |  | + |  |
