# Supplementary material for: Instruments to identify risk factors associated with adverse childhood experiences for vulnerable children in primary care in low- and middle-income countries: A systematic review and narrative synthesis
Source: PLOS Glob Public Health. 2022 Oct 5;2(10):e0000967. doi: 10.1371/journal.pgph.0000967 (PMC10021915; doi:10.1371/journal.pgph.0000967)
Supplement: S4 Table — (DOCX) [file pgph.0000967.s005.docx]

S4 Table The instrument's feasibility, biopsychosocial dimension, and applicability for use in frontline clinical settings

| Author and year | Name of the tool | Biopsychosocial dimension | Health risks addressed | Rater/time | Scaleability | Feasibility | Practicality for use in health care settings |
| --- | --- | --- | --- | --- | --- | --- | --- |
| Betancourt et al. 2014 | African Youth Psychosocial Assessment Instrument (AYPA) | Psychological | Adversities associated with political war and ethnic conflicts | Children  (30-45 Minutes) | Good for use in ethnic conflicts and disaster settings | Available for free use. | They are designed for self-use in child assessment, and it also requires a long time which may not be not feasible for busy health care settings. |
| Jordan's et al. 2008 | Child Psychosocial Distress screener (CPDS) | Psychological | Mental health effects of war | Children/ Teacher  (1-3 minutes) | Good for use in community settings to assess child psychosocial distress | Available for use in community settings to identify children in need of treatment after being involved in conflicts. | Designed for use in schools and in communities. |
| Van de Heuvel et al. 2017 | Malawi Developmental Assessment Tool (MDAT) | Physical | Poverty | Health care professionals (35 Minutes) | It has been created to assess childhood development to identify children neuro- disabilities | Available to use for free. | It can only be used for children from age (0-5)years only. |
| Su Lyn Corcoran & Joanna Wakia, 2016 | Child Status Index | Psychosocial | Poverty | Community volunteers (40 Minutes) | It was created to assess household socio-economic capacities. | Available online. | Limited for use in home-based care and specifically based on individual countries' national surveys. |
| Collings et al. 2013 | Developmental Trauma Inventory  (DTI) | Psychological | Poverty and violence | Children  (30 Minutes) | It can be used to identify the individual potential for Cognitive Behavioural therapy. | The tool is still in the developmental stages. | Limited for use in the research environment. |
| ILO - International  Labour Organisation  2014 | IPAC:  The Instrument for Psychosocial Assessment for Child Workers | Psychosocial | Poverty | Researchers  (45 Minutes) | Measure health-related quality of lie. | Available for use in the countries after validation of the instrument. | It can be long for use in the busiest health care setting. |
