# Supplementary material for: Instruments to identify risk factors associated with adverse childhood experiences for vulnerable children in primary care in low- and middle-income countries: A systematic review and narrative synthesis
Source: PLOS Glob Public Health. 2022 Oct 5;2(10):e0000967. doi: 10.1371/journal.pgph.0000967 (PMC10021915; doi:10.1371/journal.pgph.0000967)
Supplement: S5 Table — (DOCX) [file pgph.0000967.s006.docx]

S5 Table Measurement instrument to assess biopsychosocial indicators related to poverty

| Indicators | African Youth Psychosocial Assessment Instrument (AYPA) | Child Psychosocial Distress Screener (CPDS) | Malawi Developmental Assessment Tool (MDAT) | Child Status Index | Developmental Trauma Inventory  (DTI) | IPAC: The Instrument for Psychosocial Assessment for Child Workers | HIV Stigma-by-Association Scale for Adolescents | Strengths and Difficulties Questionnaire (SDQ) | WHOQOL-BREFF |
| --- | --- | --- | --- | --- | --- | --- | --- | --- | --- |
| Thinking/learning/ concentration | v | v | v | v |  | v |  | v | v |
| Malnourishment | v |  |  | v |  |  |  |  | v |
| Lack of appetite | v |  |  |  |  | v |  |  | v |
| Sadness | v | v |  | v | v | v | v | v | v |
| Food insecurity | v |  |  | v |  | v |  |  |  |
| Displacement |  | v |  |  |  | v |  |  |  |
| Depression |  | v |  |  |  |  |  |  |  |
| Withdrawal | v | v |  |  | v | v | v |  |  |
| Infection |  |  |  |  |  | v |  |  |  |
| Anxiety | v | v |  | v |  | v |  | v |  |
| Alcoholism | v |  |  |  |  |  |  |  |  |
| Sleep problems | v | v |  |  |  | v |  |  | v |
| Addiction |  |  |  |  |  |  |  |  |  |
| Homelessness/Shelter |  | v |  | v | v | v |  |  | v |
| Child labor |  |  |  |  |  | v |  |  |  |
| Loss of interest | v | v |  | v |  | v |  |  |  |
| Fatigue | v |  |  |  |  | v | v | v | v |
| Fear | v | v |  |  |  | v |  |  |  |
| Burns |  |  |  | v | v |  |  |  |  |
