# Supplementary material for: Instruments to identify risk factors associated with adverse childhood experiences for vulnerable children in primary care in low- and middle-income countries: A systematic review and narrative synthesis
Source: PLOS Glob Public Health. 2022 Oct 5;2(10):e0000967. doi: 10.1371/journal.pgph.0000967 (PMC10021915; doi:10.1371/journal.pgph.0000967)
Supplement: S6 Table — (DOCX) [file pgph.0000967.s007.docx]

S6 Table (Proposed for) rapid assessment of the biopsychosocial dimension of childhood adversity associated with poverty

| Indicators proposed | African Youth Psychosocial Assessment Instrument (AYPA) | Child Psychosocial Distress Screener (CPDS) | Malawi Developmental Assessment Tool (MDAT) | Child Status Index | Developmental Trauma Inventory (DTI) | IPAC: The Instrument for Psychosocial Assessment for Child Workers | HIV Stigma-by-Association Scale for Adolescents | Strengths and Difficulties Questionnaire (SDQ) | WHOQOL-BREFF |
| --- | --- | --- | --- | --- | --- | --- | --- | --- | --- |
| Thinking/learning/ concentration | v | v | v | v | - | v | - | v | v |
| Sadness | v | v | - | v | v | v | v | v | v |
| Withdrawal | v | v | - | v | v | v | v | - | - |
| Anxiety | v | v | - | v | - | v | - | v | - |
| Homelessness/Shelter | - | v | - | v | v | v | - | - | v |
| Isolation | v | v | - | v | v | v | v | v | v |
| Income | - | - | v | v | v | v | - | - | v |
| Forgetful | v | - | - | - | - | v | v | v | v |
| Access to healthcare | v | - | - | v | v | v | - | - | v |
| Fatigue |  |  |  |  |  |  |  |  |  |
